# Gene regulatory network analysis reveals differences in site-specific cell fate determination in mammalian brain

**DOI:** 10.3389/fncel.2014.00437

**Published:** 2014-12-18

**Authors:** Gökhan Ertaylan, Satoshi Okawa, Jens C. Schwamborn, Antonio del Sol

**Affiliations:** ^1^Computational Biology, Luxembourg Centre for Systems Biomedicine, University of LuxembourgBelval, Luxembourg; ^2^Developmental and Cellular Biology, Luxembourg Centre for Systems Biomedicine, University of LuxembourgBelval, Luxembourg

**Keywords:** SGZ, SVZ, gene regulatory network, neurogenesis, neural stem cells, differentiation, NSC

## Abstract

Neurogenesis—the generation of new neurons—is an ongoing process that persists in the adult mammalian brain of several species, including humans. In this work we analyze two discrete brain regions: the subventricular zone (SVZ) lining the walls of the lateral ventricles; and the subgranular zone (SGZ) of the dentate gyrus (DG) of the hippocampus in mice and shed light on the SVZ and SGZ specific neurogenesis. We propose a computational model that relies on the construction and analysis of region specific gene regulatory networks (GRNs) from the publicly available data on these two regions. Using this model a number of putative factors involved in neuronal stem cell (NSC) identity and maintenance were identified. We also demonstrate potential gender and niche-derived differences based on cell surface and nuclear receptors via *Ar*, *Hif1a*, and *Nr3c1*. We have also conducted cell fate determinant analysis for SVZ NSC populations to Olfactory Bulb interneurons and SGZ NSC populations to the granule cells of the Granular Cell Layer. We report 31 candidate cell fate determinant gene pairs, ready to be validated. We focus on *Ar*—*Pax6* in SVZ and *Sox2*—*Ncor1* in SGZ. Both pairs are expressed and localized in the suggested anatomical structures as shown by *in situ* hybridization and found to physically interact. Finally, we conclude that there are fundamental differences between SGZ and SVZ neurogenesis. We argue that these regulatory mechanisms are linked to the observed differential neurogenic potential of these regions. The presence of nuclear and cell surface receptors in the region specific regulatory circuits indicate the significance of niche derived extracellular factors, hormones and region specific factors such as the oxygen sensitivity, dictating SGZ and SVZ specific neurogenesis.

## Introduction

Neurons constitute a specific cell lineage in mammals. Vast majority of them arise during embryonic development, they do not undergo replicative aging and new neurons are generated from neural stem cells (NSCs) through a process called neurogenesis only in specific regions in the adult mammalian brain (Altman and Das, 1965; Lois and Alvarez-Buylla, 1993; Lois et al., 1996; Gage et al., 1998). Cell divisions during neurogenesis can be either symmetric or asymmetric. Symmetric division can increase the pool of stem cells when they lead to two new stem cells, while terminal symmetric division leads to the generation of two neurons and a depletion of the stem cell pool (Doetsch et al., 1997, 1999; Palmer et al., 2000). Asymmetric mitosis of NSCs produces one NSC and one neural progenitor cell (NPC), daughter cell with limited differentiation capacity for neuronal or glial lineages (Gage, 2000; Temple, 2001). Extracellular factors and brain region specific transcription factors (TFs) are believed to be essential for NSC maintenance and proliferation which include epidermal growth factor (EGF) (Gritti et al., 1999; Tropepe et al., 1999), fibroblast growth factor (FGF) (Kalyani et al., 1997; Raballo et al., 2000), Sonic Hedgehog (Shh) (Wechsler-Reya and Scott, 1999) and Wnt signaling (Ikeya et al., 1997).

Two major sites of adult neurogenesis reported to date are the subgranular zone (SGZ) of the hippocampal dentate gyrus (DG) and the subventricular zone (SVZ) lining the lateral ventricles (Lois and Alvarez-Buylla, 1993; Gage et al., 1998). Although both sites harbor NSCs, the differentiation process is site specific and results in different types of cells. The SVZ has the ability to produce neurons, glia and oligodendrocytes (Rao and Mayer-Proschel, 1997; Rao, 1999; Alvarez-Buylla et al., 2001), while the SGZ specific stem and progenitor cells are able to produce granule neurons and astrocytes (Seri et al., 2001; Fukuda et al., 2003). Given the paramount importance of natural reservoirs of NSCs in neurodegenerative diseases, the site-specific regulatory mechanisms acting in SGZ and SVZ are surprisingly not well understood.

The niche of the SGZ in the DG of the hippocampus consists of populations of stem and progenitor cell types, which have distinct and variable cell division and survival rates. Quiescent NSCs have a single radial process that extends through the granule cell layer (GCL) and express markers such as glial fibrillary acidic protein (GFAP) and Nestin (Seri et al., 2001; Fukuda et al., 2003). Another cell type is the horizontal cell, which divides more rapidly; however, the exact lineage association between radial and horizontal cells is still not established. Quiescent NSCs leave their dormant state and proliferate; they divide to produce transit-amplifying progenitors (TAPs) that have the potential to differentiate into neurons and astrocytes. The immature neurons produced from TAPs have the potential to differentiate to give rise to mature glutamatergic granule neurons in the GCL (Cameron et al., 1993; Kempermann et al., 2004). During this process intra- and intercellular signals arising from the microenvironment, including cellular components (vascular and glial cells, and granule neurons in the environment) and non-cellular components (extracellular matrix proteins and secreted molecules), have an effect on the activity of SGZ NSCs as well as the differentiation and survival capacity of the immature neurons along the SGZ GCL axis (Palmer et al., 2000; Ma et al., 2005, 2009; Morrens et al., 2012). Furthermore, both astrocytes and neurons differentiated from the TAPs are reported to play an instructive role to promote NSC self-renewal and differentiation. This naturally creates the feedback loop to maintain homeostasis in the microenvironment. Another lines of evidence indicate that neural progenitor cells respond to neuronal activity in the form of glutamate and GABA secretion as part of their differentiation program (Song et al., 2002; Deisseroth et al., 2004; Tozuka et al., 2005). In addition to these neurotransmitters, astrocytes are described also a potential source of niche factors such as Notch, Shh, bone morphogenetic proteins (BMPs), and Wnts (Ahn and Joyner, 2005; Lie et al., 2005; Ables et al., 2010; Mira et al., 2010).

The second region in which neuronal production persists throughout life in mammals is within the SVZ of the lateral ventricle. Earlier studies have shown that a subset of radial glial cells subsequently give rise to astrocyte-like NSCs that can serve as quiescent stem cells of the SVZ during postnatal and adult stages (Merkle et al., 2004; Spassky et al., 2005). In addition to their reported morphological resemblance to astrocytes, type B1 cells also express markers like GFAP, glutamate transporter (GLAST) and brain–lipid-binding protein (BLBP) (Doetsch et al., 1999; Nomura et al., 2010). Type C cells, the progeny of type B cells, rapidly proliferate and are often reside in clusters near blood vessels indicating possible niche-provided factors for their survival (Kriegstein and Alvarez-Buylla, 2009). Similar to type 2 TAPs of the SGZ, type C cells have elevated levels of *Dlx2*, *Ascl1*, and *Pax6* (Brill et al., 2008). Finally, those type C cells give rise to neuroblasts, which form migratory pathways enclosed within astrocyte tubes, the so called rostral migratory stream reaching from the SVZ to the olfactory bulb (Lois et al., 1996). These multiple progenitor cell types ultimately give rise to a diverse array of olfactory bulb (OB) interneurons, including deep granule interneurons and superficial granule interneurons (Merkle et al., 2007; Lledo et al., 2008). Neurons of the OB are mostly GABAergic interneurons; however, fate-mapping study suggested the existence of glutamateric- and dopaminergic neurons (Brill et al., 2009) as well. Together, current studies support the model of “microenvironment supported adult neurogenesis,” in which NSCs are regulated by microenvironment derived signals, allowing their proliferative expansion and differentiation into mature cell types in SGZ and in SVZ. Ultimately, niche-derived signals are relayed to the NSC genome to control transcription of genes involved in self-renewal, differentiation and survival. The detailed review including of SGZ and SVZ neurogenesis is found elsewhere (Hsieh, 2012).

At the molecular level, region specific roles of the TFs, cell surface markers as well as extracellular factors constitute a distinct microenvironment in SGZ and SVZ resulting in reported differences in cell lineage commitment such as the propensity to differentiate into granule cells of the GCL and interneurons of the OB respectively.

The role of TFs determining cell fates, known as lineage specifiers, is one of the important concepts emerged from studying stem cell differentiation. A simple model considers that two lineage specifiers, which repress each other and activate themselves, are responsible for binary cell fate decisions. Several examples of these binary cell fate choice mechanisms have emerged in the last decade. A more general view postulates that stem/progenitor states, corresponding to metastable states, are maintained by the balanced expression of two rival lineage specifiers, which compete for differentiation into mutually exclusive cell fates (Huang et al., 2006; Roeder and Glauche, 2006). Moreover, disturbance of this equilibrium leads to cell fate differentiation.

In this manuscript we performed a comparative transcriptomics analysis between the mouse SGZ and SVZ in order to investigate region specific gene expression profiles, including TFs, receptors and extracellular factors. Moreover, gene regulatory networks (GRNs) of differentially expressed TFs between these two regions were constructed using a method that we have previously introduced (Crespo et al., 2013). Further, key strongly connected components (SCC) belonging to each region-specific GRN were identified. Indeed, SCCs have been shown to be important GRN stability motifs that contain genes responsible for maintaining cell identity (Crespo and Del Sol, 2013). Finally, based on the notion that two rival cell fate determinants exhibit a significantly disbalanced expression pattern in the differentiated cells in comparison to the stem/progenitor cell, we identified several pairs of these TFs belonging to SCCs as candidates of cell fate determinants in these regions.

Our results allow us to highlight potential cell fate determinant pair candidates and their site specificity in regions of neurogenesis. These pairs constitute a good starting point for future studies addressing cellular reprogramming and transdifferentiation for neurodegenerative disorders.

## Materials and methods

### Gene expression data

The gene expression microarray data for SGZ and GCL are obtained from NCBI Gene Expression Omnibus (GEO) under accession number GSE39697 (Edgar et al., 2002). In this study authors explain that brains from 10 to 11-week-old C57BL/6 male mice (*n* = 9) were quickly removed, flash-frozen in OCT (Sakura Finetek) and stored at −80°C. Cryostat sections (12 μm) were stained with Cresyl Violet and rapidly dehydrated through graded xylenes. An Arcturus PixCell II machine (Arcturus) was used to isolate two- to three-cell thick bands from the outer and inner (SGZ, and specifically excluding cells of the hilus) portions of the dentate GCL (Miller et al., 2013). The RNA purification, amplification and microarray hybridization (to Affymetrix MG-U74Av2 arrays) experiments are performed according to the protocols in house. Explanation of the animal procedures, RNA purification, amplification and microarray hybridization can be found in detailed elsewhere (Miller et al., 2013).

The RNASeq data for SVZ is obtained from NCBI GEO under accession number GSE45282 (Ramos et al., 2013). In this study the brain from adult (older than postnatal day 60) male C57BL/6 mice was removed from the skull and placed in the ice-cold L15 media and a coronal slab (0.5-mm-thick) was obtained. The lateral SVZ and striatum were then micro dissected, avoiding contamination from the corpus callosum. FACS sorting of the SVZ cells was performed as described earlier (Pastrana et al., 2009). DG region was microdissected with a Vibratome and 300-mm-thick coronal sections obtained are placed in ice-cold L15 media. The high-throughput sequencing (Illumina) and sequencing data processing and mouse genome mapping is performed according to protocols as explained in detail in Ramos et al. (2013).

The background gene expression signal for detecting over/under expression for predicting pairs has been obtained from GEO under accession number GSE2882 (Sugino et al., 2006). In this study authors have characterized 12 populations of neurons from the mouse forebrain. These regions include hippocampus, cingulate cortex, somatosensory cortex, amygdala and thalamus. The data consists of 36 samples from 12 different cell types with three biological replicates (each sample from a different animal). The animals were all adult male mice, 57–106 day old with varying genetic background (The contribution of C57BL/6J ranged from 31 to 100%, with an average of 67 ± 26% (mean ± SD) (Sugino et al., 2006). The median expression per gene calculated from this data has served as the basal expression level per gene for the mouse forebrain.

### *In situ* hybridization

The ISH data presented herein is obtained from Allen Brain Atlas portal (http://www.brain-map.org/). The *in situ* hybridization shown in this manuscript is from the Allen Mouse Brain Atlas. All technical details describing this project is available under the “Documentation” tabs in the atlas (http://mouse.brain-map.org/static/atlas).

### Microarray data normalization, discretization and analysis

Gene expression data from GSE3997, GSE45282, and GSE2882 have been merged and used for downstream analysis. The in-group normalization of the data has been performed in R. BioConductor “affy” package is used for reading Affymetrix microarray data and summarizing the probe level data into Robust Multichip Analysis (RMA) expression measures (Bolstad et al., 2003). The FPKM converted values of RNASeq data from GSE45282 has been used for downstream analysis.

#### Quartile expression

Equal Frequency Discretization (EFD) divides the sorted E(n,:) into k intervals so that each interval contains approximately the same number of expression values. We have extended the Equal Frequency Discretization method (EFD) to discretize and calculate the differential expression between SGZ, GCL, SVZ, and OB expression data. This discretization allows us to assign expression values to each gene between 1 and 4 depending on the relative abundance (Quartiles 1–4) of the transcripts (mRNA, lncRNA, miRNA etc.) in each dataset. We have used the median values for summarizing different probes for the same gene product. We have used the median values also per gene when there are multiple replicates were available (in SGZ, GCL, and SGZ). The choice of the median values was motivated by choosing a robust discretization to avoid the outlier effects. Hence increase the sensitivity of the differential expression calculation.

We have calculated the Quartile Expression (QE) for each gene by evaluating the quartile change of the gene in two experimental conditions. Therefore, in this comparison differentially expressed is defined as the change in the relative abundance (or quartile expression) of the gene in two different regions. We have defined minimum two (±2) difference as statistically significant to increase sensitivity to signal ratio by avoiding the possible boundary conditions around the quartiles. We have calculated QE for SGZ vs. SVZ, SGZ vs. GCL, and SVZ vs. OB (Supplementary Table).

**Figure 1:**
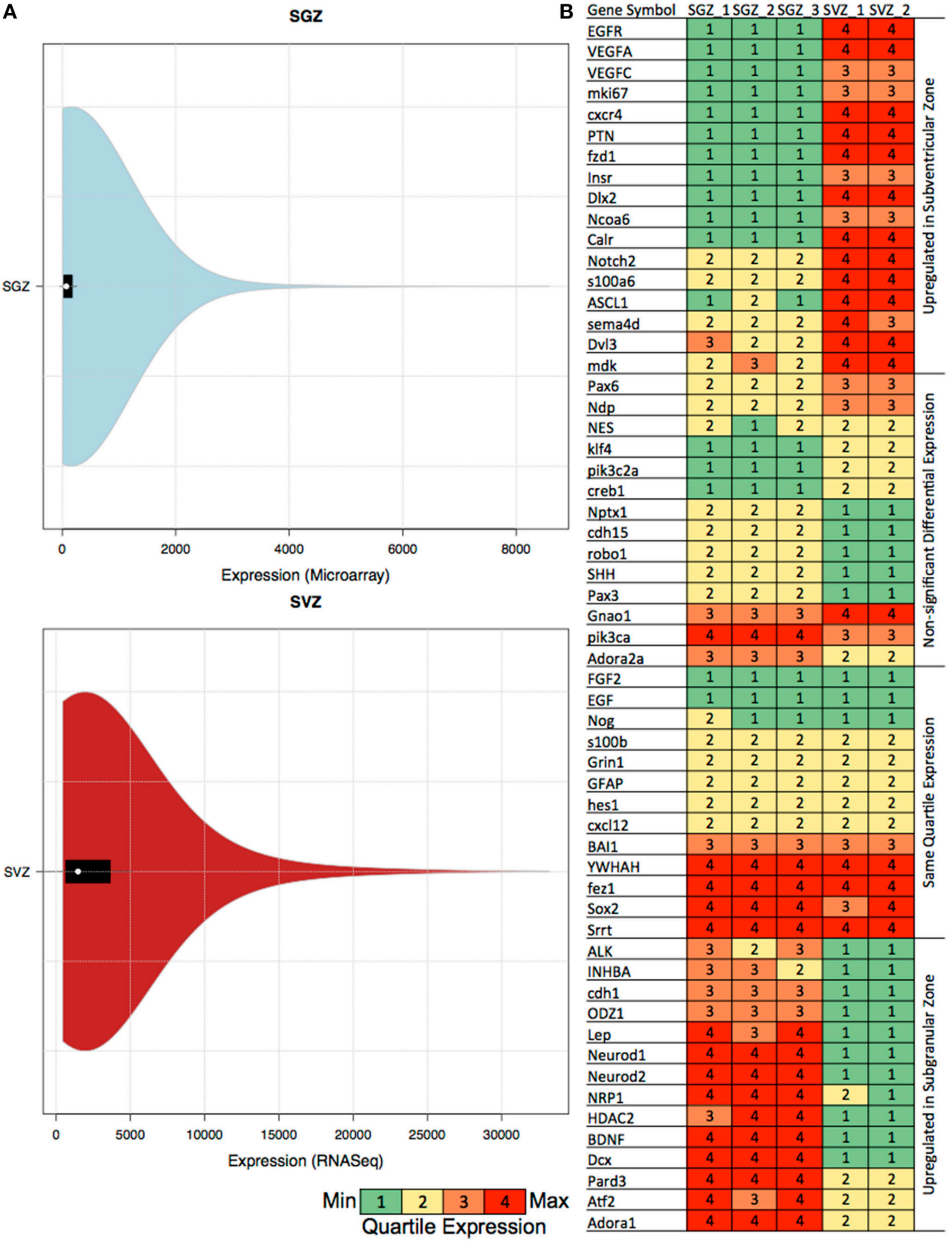
**Table showing the quartile expression of genes associated with neurogenesis and the distribution of gene expression in SGZ and SVZ. (A)** Violin plots showing the density trace with the box plot of gene expression in SGZ (microarray expression intensity) and SVZ (gene-level signal intensity) values. The black region in each plot is the box plot with white dot with the mean and the upper and lower bounds of the plots are the upper and lower quartiles. As shown in the figures, even though the absolute values are different, the violin plot distribution of the expression in each region is very similar, hence rendering the comparison via quartile expression viable. **(B)** The genes associated with neurogenesis are clustered in four clusters according to their differential expression in SGZ and SVZ: Upregulated in Subventricular Zone, Non-significant Differential Expression, Same Quartile Expression and Upregulated in Subgranular Zone. The quartile expression is color coded as shown below green, yellow, orange, and red indicating low, low-medium, high-medium and high expression respectively.

We have also calculated the distribution of the gene expression skewness (SGZ: 6.4–7.18 and SVZ: 2.87–2.89) and kurtosis (SGZ: 64.1–76.9 and SVZ: 10.6–10.8) per region (in SGZ and SVZ) for examining the suitability of our approach. The quartile expression values for the previously associated genes, and violin plots including all experiments for both SGZ and SVZ are shown in Figure 1B.

### Gene ontology analysis

For Gene Ontology (GO) analysis we have used funcassociate (Berriz et al., 2009) to investigate key factors, pathways and regulatory mechanisms that are responsible for the maintenance and differentiation of NSC identities in mammalian brain.

We have employed a strict criterion for performing GO analysis for differentially expresses genes between SGZ and SVZ. Only GO processes with adjusted *P*-value below 0.1% (P_adj ≤ 0.001), log-odds ratio over 0.3 (LOD ≥ 0.3) are considered significant. We have sorted the results for number of genes in each associated GO term (X). For presentation in in Table 1, we selected top 14 GO terms that have minimum number of genes per GO term for specificity.

**Table 1:** **Gene ontology annotations for differentially expressed genes between SGZ and SVZ**.

**DE genes**	**GO genes**	**Log-odds ratio**	Gene category^*^
52	1521	0.39	**Nervous system development [GO:0007399]**
49	1392	0.40	Negative regulation of cellular metabolic process [GO:0031324]
38	939	0.46	**Neuron differentiation [GO:0030182]**
24	437	0.59	**Brain development [GO:0007420]**
30	667	0.50	*Protein complex binding [GO:0032403]*
48	1416	0.38	Negative regulation of macromolecule metabolic process [GO:0010605]
26	543	0.53	Chromosomal part [GO:0044427]
39	1052	0.42	*Protein dimerization activity [GO:0046983]*
37	983	0.42	Negative regulation of nucleobase-containing compound metabolic process [GO:0045934]
40	1112	0.40	**Neurogenesis [GO:0022008]**
38	1040	0.41	Negative regulation of cellular biosynthetic process [GO:0031327]
37	1000	0.42	Negative regulation of nitrogen compound metabolic process [GO:0051172]
38	1044	0.41	**Generation of neurons [GO:0048699]**
21	396	0.57	*Protein binding transcription factor activity [GO:0000988]*

* For all processes the adjusted p-value is smaller or equal to 0.10%. Gene categories are sorted in ascending order for the adjusted p-values (not shown) and categories associated with neurogenesis are shown in bold.

We have used the package funcassociate (Berriz et al., 2009) that statistically identifies the terms that were associated with differentially expressed genes (defined by adjusted *p* ≤ 0.1 and LOD ≥ 0.3).

### Gene regulatory network construction, contextualization and analysis

Human direct gene interactions were retrieved from MetaCore from Thompson Reuters [GeneGo Inc. (https://portal.genego.com/)] to infer biological relationships and associations that have been extracted from the biomedical literature relevant for these sites.

The interaction types “Transcription regulation” and “Binding” were kept for the subsequent analyses. Pruning of network edges (interactions) was performed using the method developed by Crespo et al. (2013), which was implemented in MATLAB using the genetic algorithm (ga) function. This algorithm is designed with the assumption that each cellular phenotype is a stable steady state (attractor) of the GRN. The algorithm removes edges that are inconsistent with the Booleanized gene expression data, thereby obtaining a network not only based on previous knowledge but also customized for a given biological condition. The Boolean simulation was carried out using the pbn-matlab-toolbox (http://code.google.com/p/pbn-matlab-toolbox/downloads/list) using the synchronous updating scheme. The logic rule was defined, so that the number of activating edges and inhibiting edges acting on a gene were compared and the one with a higher number dominated. For example, if a gene had three activating edges and two inhibiting edges acting on it, the activation logic was used in the next update. If both numbers were the same, the inhibition logic was set to dominate. During this process, “unassigned” interactions (i.e., interactions without knowledge of activation or inhibition) were randomly assigned “activation” or “inhibition” and the one that yielded a better result was taken. The contextualized network was loaded in Cytoscape (version 2.7.0) (Shannon et al., 2003). The network stability motif, strongly connected component (SCC), was identified using the Cytoscape BiNoM (version 2.5) plugin (Zinovyev et al., 2008).

### Cell fate determinant pairs analysis

The difference in the expression ratio of a pair of genes between the stem/progenitor cell and differentiated cell was computed by

$$\left(gene1_{differentiated}-gene2_{differentiated}\right)-\left(gene1_{stem}-gene2_{stem}\right)$$

where *gene*1 and *gene*2 indicate the log2 median gene expression value over biological replicates. The suffix “differentiated” indicates the differentiated cell types (i.e., GCL or OB) and “stem” indicates the stem/progenitor cell types (i.e., SGZ and SVZ). This value was calculated for all pairs of TFs and statistically significant pairs were detected by a robust *z*-test. The values were first normalized by their expression ratios of the stem/progenitor cells and the z-score was computed after trimming the 2.5% outliers on both sides. The z-score of the trimmed data points was extrapolated from the z-score function. The z-score was then converted into the *p*-value. The *p*-value was corrected for multiple testing by the Benjamini-Hochberg method. Pairs with adjusted *p*-value below 0.05 were kept. Additional criteria were set to narrow down candidate cell fate determinants [Supplementary Figure S3 (flowchart)]. These criteria were (1) one of the genes in a pair has to be differentially up-regulated in the differentiated cell type (i.e., GCL and OB), (2) both genes in a pair are not differentially down-regulated in the stem/progenitor cells (i.e., SGZ and SVZ), and either (3) both genes in a pair are directly connected in the SCC or (4) one of the genes in a pair is present in the SCC and the other gene is not present in the SCC but is known to directly interact with the other gene.

## Results

The neurogenic potential of the SVZ along the walls of the lateral ventricle and the SGZ of the DG is well established. Each region has been studied in isolation that resulted in our understanding of the mammalian neurogenesis *in-vivo*. Naturally gene ontologies (such as neurogenesis; GO:0022008, nervous system development; GO:0007399, regulation of nervous system development; GO:0051960) have been created from these studies. This equipped us with tools to identify neurogenic gene expression signatures from other processes of the neural system.

Recent studies indicate the differences between the SGZ and SVZ, site-specific regulation of these regions by molecular cues arising from their complex heterogeneous cellular environment and their differential contribution in generating various cell types in the adult mammalian brain.

For the molecular annotation and functional analysis of NSC in two regions in the adult mammalian brain we have merged and analyzed genome-wide gene expression of SGZ and GCL from Miller et al. (2013) and SVZ and OB from Ramos et al. (2013).

### SGZ and SVZ differ in expression of factors associated with neurogenesis

We have composed the specific markers and genes previously associated with neurogenesis to the best of our knowledge. The list of associated factors with their references are shown in the Supplementary Tables under Genes associated and Present tab.

To be able to compare GRNs we have extended the Equal Frequency Discretization method (EFD) to calculate the Quartile Expression (QE) for each gene. This discretization allows us to assign expression values to each gene between 1 and 4 depending on the relative abundance in the experiment, therefore in this comparison differentially expressed is defined as the change in the relative abundance (or Quartile Expression) of the gene in two different regions. We have also calculated the distribution of the gene expression, skewness and kurtosis per region (SGZ and SVZ) for characterizing our method (See Materials and Methods). The distributions of gene expression for each region show very similar patterns as shown by the violin plots in Figure 1A, hence allowing our method to capture the essential differences in gene expression between SGZ and SVZ.

There were 1578 genes differentially expressed (having a differential quartile expression QDE ≤ −2 or QDE ≥ 2) between two regions among which 828 of them were up-regulated in SVZ and 750 of them were up-regulated in SGZ.

The quartile expression values for the previously associated genes for each region are shown in Figure 1B. In depth analysis of the quartile expression of these genes indicates four clusters: (i) Up-regulated in SGZ, (ii) Up-regulated in SVZ, (iii) Same quartile expression, and (iv) Non-significant differential expression.

One of the stark differences is observed in the Vascular Endothelial Growth Factor (VEGF) genes (*Vegfa* and *Vegfc*). *Vegf* over-expression is associated with increased neurogenesis earlier with SVZ and SGZ neurogenesis (Jin et al., 2002; Warner-Schmidt and Duman, 2007) and reviewed in detail (Fournier and Duman, 2012; Mackenzie and Ruhrberg, 2012). Although the link with *Vegf* genes and proliferation-linked neurogenesis is established, region specific effects and the possible role in determining cell fate is not explored.

The overexpression of the homeobox protein *Dlx2* and the EGF receptor (*Egfr*) are observed to be SVZ specific in our analysis. This finding overlaps with the reported SVZ specific interaction between two genes, *Dlx2* promoting the lineage transition from NSCs to TAPs and at the same time enhancing the proliferative response of neuronal progenitors to *Egf* (Suh et al., 2009).

Another important factor *Notch2*, whose down-regulation is implicated in subsequent NSC differentiation to astroglial lineage (Tchorz et al., 2012), is observed to be upregulated in SVZ with respect to SGZ. This is in line with the astrocytic features associated to SVZ NSCs.

*Neurod* is very well established as a transcription factor required for maintenance of neurogenesis and differentiation of the granule cells in the cerebellum and hippocampus (Miyata et al., 1999). The essential role of NeuroD1 in the differentiation and survival of neuronal precursors is stressed by Gao et al. (2009). The elevated Neurod1 and Neurod2 expression in SGZ is possibly due to the different spatial scales of neurogenesis and differentiation for SGZ and SVZ (SGZ to GCL being more compact and SVZ to OB being spread out including RMS).

*Sox2* is essential for maintenance of neural stem cell fate and as expected is expressed in the last (4th) quartile in both SGZ and SVZ regions. GFAP, another widely used marker of quiescent NSC, is expressed in both SGZ and SVZ.

### SGZ and SVZ differ in classes of differentially expressed genes

We next sought to measure the extent to which the gene expression profiles of the neurogenic niche are conserved between SGZ and SVZ and also determine the functional classes of the genes distinguishing different regions. We have performed an over-representation analysis using the Gene Ontology (GO) associations. GO terms are nested functional categories that summarize the known molecular functions, biological processes and cellular compartments associated with each gene. Enriched GO terms for genes that are differentially expressed between SGZ and SVZ are shown in Table 1 (Adjusted *p* ≤ 0.1%).

Given the neurogenic potential of both regions, we expect to find only marginal differences in GO analysis, possibly related to the specific differentiation potential of each zone. However, over represented biological processes that differentiate gene expression of both regions include five categories associated with neurogenesis, namely: Nervous system development, Neuron differentiation, Brain development, Neurogenesis and Generation of Neurons. This is striking, because significant portion of stem cell maintenance and cellular proliferation markers (such as *Sox2* and *Gfap*) are not differentially expressed between SGZ and SVZ. However, GO enrichment shows strong association with neurogenesis and neuron differentiation indicating *possible involvement of niche specific factors*. This leads to the conclusion that in addition to the mutual and comparable expression of stem cell factors such as *Sox2*, *Oct4*, *Klf4*, *Myc*, and *Nanog*, the genes associated with neurogenesis are not expressed uniformly in SGZ and SVZ, leading to diverse differentiation potentials in each region.

It is also important to note that three molecular functions, protein complex binding, protein dimerization activity and protein binding transcription factor activity, are over represented together suggesting potential differences on the protein function level.

### SGZ and SVZ have distinct regulatory mechanisms

**Figure 2:**
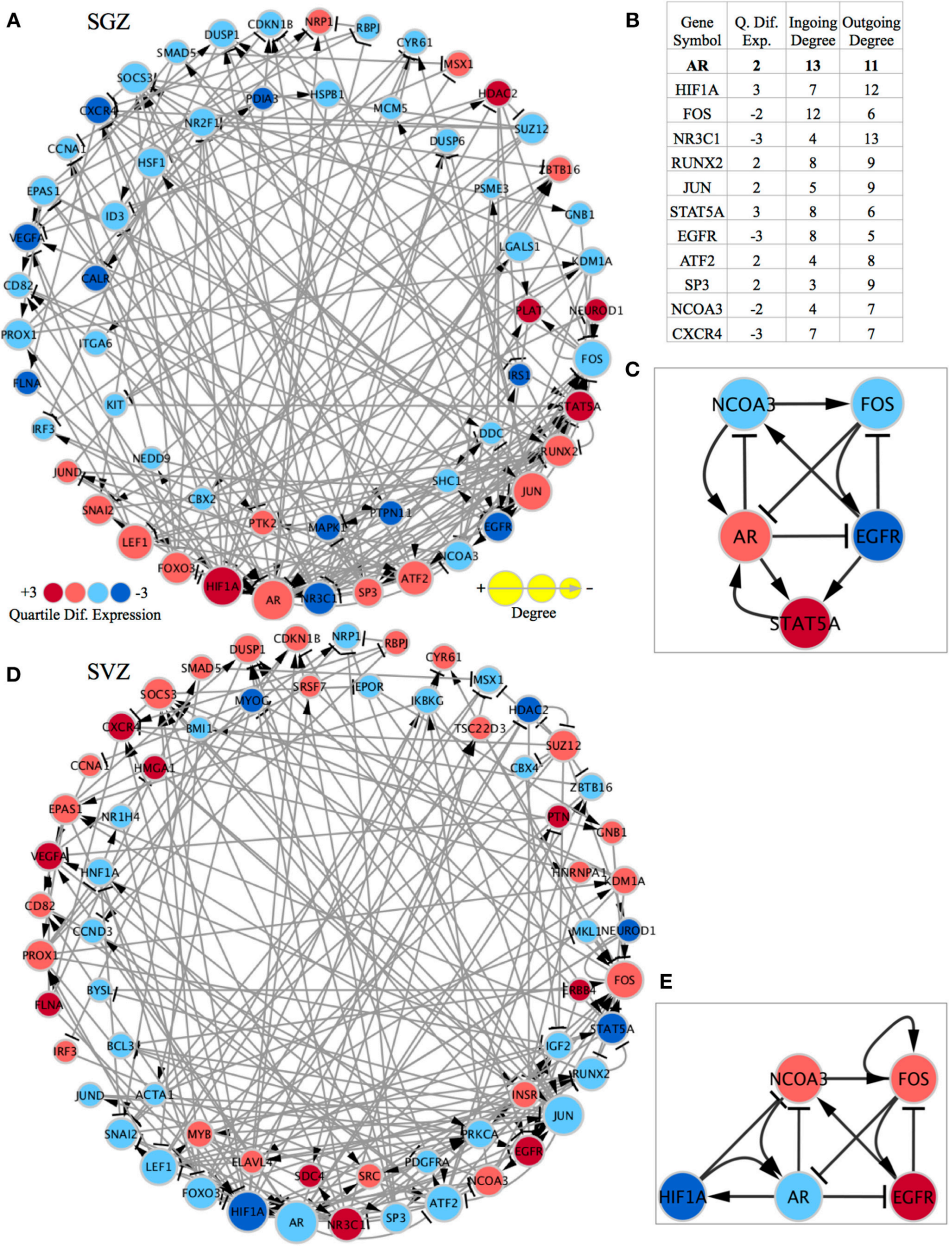
**Region specific gene regulatory networks and sub-networks and gene list showing the importance of highly connected genes.** The quartile differential expression of each gene in the network is color-coded below showing the difference in quartile expression with respect to the other region. Red indicates significantly higher (+3), pink indicates higher (+2), light blue indicates lower (−2), dark blue indicates significantly lower differential quartile expression in the region of interest vs. the other region. The size of each node is size coded to indicate the degree of this gene in the network as displayed below. The standard graphical notation for biological networks is used arrow heads indicating activation/up-regulating and bar-headed arrows indicating inhibition/down-regulating interactions. **(A)** SCC of the SGZ specific regulatory network: The outer ring consists of genes that are shared with the SCC of the SVZ specific network and have differential quartile expression. The inner ring consists of genes only present in the SCC of the SGZ regulatory network (their expression is important only in SGZ regulatory network and not in SVZ). **(B)** The table showing the quartile expression, ingoing & outgoing degrees for highly connected genes in the shared genes (outside ring genes) in both networks. The list of genes and their order of importance is very similar in SGZ and SVZ specific networks (the degrees in the table are from SGZ specific network and are almost identical in the SVZ specific network). **(C)** The AR contextualized SGZ specific SCC sub-network showing the interconnected nature and the regulation of factors around AR. **(D)** SCC of the SVZ specific regulatory network: The outer ring consists of genes that are shared with the SCC of the SGZ specific network and have differential quartile expression. The inner ring consists of genes only present in the SCC of the SVZ regulatory network (their expression is important only in SVZ regulatory network and not in SGZ). **(E)** The AR contextualized SVZ specific SCC sub-network showing the interconnected nature and the regulation of factors around AR. Note that FOS has a self-activation and STAT5A is replaced by HIF1A in this network for representation purposes.

In order to identify differences between the SGZ and SVZ, we discretized their genome-wide expression patterns, and calculated differentially expressed genes (DEGs) based on our criteria as explained in materials and methods. We then constructed two region specific GRNs from differentially expressed TFs using a method that we previously implemented (Crespo et al., 2013). This method removes interactions which are inconsistent with expression data and generates networks that explain cell specific gene expression programme. We previously used network strongly connected components (SCCs) as gene regulatory network (GRN) stability motifs and identified candidate genes that are responsible for the stabilization of cellular identities (Crespo and Del Sol, 2013). Therefore, we next identified SCCs in the GRNs (Figures 2A,D). The SCC for the SGZ had 58 genes (17 are up and 41 are down-regulated in SGZ), whereas the SCC from the SVZ had 63 genes (31 are up and 32 are down regulated in SVZ). Both SCCs had 37 genes in common with the same predicted gene expression state including *Ar*, *Hif1a*, *Foxo3*, *Lef1*, *Jun*, *Jund*, *Fos*, *Snai2*, *Nr3c1*, *Sp3*, and *Atf2*. Additionally 21 and 26 of those genes are unique to the SGZ and SVZ, respectively, revealing the similar and distinct gene regulatory mechanisms that stabilize the NSCs in these regions. We have also focused on highly connected genes and genes that had the highest number of out-going edges within each SCC, as these genes are likely to have a high regulatory influence on the SCC. Indeed, this criterion called “out-degree interface” was previously used in our study (Crespo and Del Sol, 2013) for identifying master regulatory TFs within SCCs. Here, we have identified *Nr3c1*, *Hif1a*, and *Ar* as master regulators in this system with the three highest out-degree interfaces in both SGZ and SVZ.

To further elucidate the network dynamics in the proximity of these master regulators we have chosen *Ar* (androgen receptor) as our focus due to its highest total degree (3rd highest outgoing and 1st ingoing) and its gender specific biological implications (Figure 2B).

Subsequently, we have built SGZ and SVZ specific GRNs from AR (and all 1st neighbor genes) as explained above and the SCCs of these GRNs were identified (Supplementary Figures S1, S2 and Supplementary Networks). This has accomplished by while pruning of network edges, instead of global optimization, we have implemented optimization for edges connected to *Ar* for obtaining perfect consistency around *Ar*. The SGZ specific SCC sub-network around *Ar* is shown in Figure 2C. Network dynamics analysis in this sub-network indicates the “dominance” of AR in this subnetwork. *Ar* directly regulates all three out-degree connected nodes; inhibit *Ncoa3*, *Egfr* and forms a reinforcing feedback loop with *Stat5*. Moreover, the expression pattern observed in SGZ vs. SVZ for this sub-network corresponds to an attractor state presenting the stability of the observed expression pattern.

*Ar* is present in many of the body's tissue, where it binds to androgens (such as testosterone). Its expression is highly gender specific and the resulting androgen-receptor complex binds to DNA and regulates the activity of androgen responsive genes.

An inverse correlation between EGF and androgen signaling is reported in epithelial cells (Léotoing et al., 2007). It has been demonstrated that, cell cycle exit at G_0_ phase and the setting of the differentiation process is a prerequisite for having an active androgen signaling. Once cells have completed this process, androgen signaling is essential for the maintenance of the differentiated functions and for repressing the EGF-dependent signaling in epithelial cells.

Here we report that the balance between AR and EGFR signaling is skewed toward *Ar* up-regulation in SGZ and *Egfr* up-regulation in SVZ, indicating a possible mechanism for distinct differentiation phenotypes in those regions.

*Ncoa3* encodes for a co-activator that interacts with nuclear hormone receptors to enhance their transcriptional activity. It has histone acetyltransferase activity and considered to act via remodeling of chromatin. Involved in the co-activation of different nuclear receptors, such as for steroids (GR and ER), retinoids (RARs and RXRs), thyroid hormone (TRs), vitamin D3 (VDR) and prostanoids (PPARs). In SGZ sub-network, the up-regulation of *Ar* combined with the scarcity in EGF signaling results in down-regulation of *Ncoa3*, in turn results in the impeded support for the *Ar* expression.

The SVZ specific SCC-sub-network around *Ar* is shown in Figure 2E. Analysis of this sub-network indicates *Fos* dominance with self-activation and a negative feedback loop via *Egfr*. Furthermore, *Fos* inhibits *Ar* expression, practically rendering the *Ar* dependent inhibition of *Ncoa3* and *Egfr* obsolete.

*Hif1a* constitutes another remarkable finding significantly differentially expressed between SGZ and SVZ. *Hif1a* encodes the alpha subunit of transcription factor *Hif-1*, which functions as a master regulator of cellular homeostatic response to hypoxia by activating transcription of many genes. These include genes involved in energy metabolism, angiogenesis, apoptosis, and others whose protein products increase oxygen delivery or facilitate metabolic adaptation to hypoxia.

Mazumdar et al., have demonstrated that *Hif1a* modulates Wnt/β-catenin signaling in hypoxic embryonic stem (ES) cells by enhancing β-catenin activation and expression of the downstream effectors *Lef-1* and *Tcf-1*. They have extended this finding to primary cells, including isolated NSCs, and NOT to differentiated cells. They also report that *in vivo*, Wnt/β-catenin activity was closely associated with low O_2_ regions in the SGZ of the hippocampus. The deletion of *Hif1a* impaired hippocampal Wnt-dependent processes, including NSC proliferation, differentiation and neuronal maturation. This decline correlated with reduced Wnt/β-catenin signaling in the SGZ (Mazumdar et al., 2010).

Differential expression and regulation of *Hif1a* (up-regulated in SGZ) in our analysis is in perfect agreement and suggests diverse niches with varying O_2_ sensitivity and Wnt/β-catenin activity in SGZ and SVZ.

### Region specific cell fate determinant pairs

Previous theoretical and experimental studies indicated that two rival cell fate determinants show a balanced expression pattern in the stem/progenitor cell and breaking this balance would lead to differentiation (Huang et al., 2006; Roeder and Glauche, 2006). Assuming that this notion also holds true for neurogenesis, we next aimed to identify pairs of TFs that exhibited this expression pattern between the SGZ and GCL, and between the SVZ and OB. The goal here is to find region specific cell fate determinants, which give rise to the granule cells of the GCL and interneurons of the OB from the SGZ and SVZ, respectively. In addition, we set other criteria to narrow down candidate cell fate determinants [Supplementary Figure S3 (flowchart)]. These criteria were (1) one of the genes in a pair has to be differentially up-regulated in the differentiated cell type (i.e., GCL and OB), (2) both genes in a pair are not differentially down-regulated in the stem/progenitor cells (i.e., SGZ and SVZ), and either (3) both genes in a pair are directly connected in the SCC or (4) one of the genes in a pair is present in the SCC and the other gene is not present in the SCC but is known to directly interact with the other gene.

The results of the cell fate determinant pair analysis are shown in Table 2. For the SGZ to GCL predictions, out of 18 predicted pairs, 12 of them has at least one of the pair is associated earlier with neurogenesis while 6 pairs are novel predictions. From the SVZ to OL region predictions 9 out of 13 predictions have one or both of the pair genes have a reported link to neurogenesis.

**Table 2:** **Cell fate determinant pairs predicted for SGZ to GCL and SVZ to OB**.

**PAIR1 (P1)**	**PAIR2 (P2)**	**Adjusted *P*-value**	**Gene/Pair association with neurogenesis**
**SGZ TO GCL**			
EED	HIC1	<10^−6^	NA
SOX2	SRRT	<10^−3^	SOX2, SRRT, SOX2-SRRT Pair
ID4	TCF3 (E2A)	<10^−3^	ID4, TCF3
SOX2	NCOR1	<10^−2^	SOX2
NEUROD1	EGR1	<10^−2^	NEUROD1
SOX2	ARID2	<10^−2^	SOX2
SOX4	BATF3	<10^−2^	SOX4
NOTCH1	RUNX2	<10^−2^	NOTCH1
SP3	RELA	<10^−2^	NA
SOX2	CXXC1	<10^−2^	SOX2
TWIST1	PPARGC1A	<10^−2^	NA
NEUROD1	PCGF2	<10^−2^	NEUROD1
SP3	SP1	<10^−2^	NA
ATF3	SNAI2	<10^−2^	ATF3
ETS2	TCF7L2	<10^−2^	NA
SOX2	ZFX	<10^−2^	SOX2
SOX2	SMARCA4	<10^−2^	SOX2
ATF1	BRCA1	<10^−2^	NA
**SVZ TO OB**			
EGR2	LHX2	<10^−10^	LHX2
CITED2	CREBBP	<10^−2^	CREBBP
SOX2	FOXO1	<10^−2^	SOX2, FOXO1
CITED2	KAT2A	<10^−2^	NA
EOMES	STAT6	<10^−2^	EOMES
PAX6	PRDM5	<10^−2^	PAX6
CITED2	CARM1	<10^−2^	NA
PAX6	NFIA	<10^−2^	PAX6, NFIA
DLX2	SMAD3	<10^−2^	DLX2, SMAD3
PAX6	AR	<10^−2^	PAX6, AR
CREB3L2	NFIL3	<10^−2^	NA
NFIL3	RORC	<10^−2^	NA
GLI3	SALL1	<10^−2^	GLI3, SALL1

**Figure 3:**
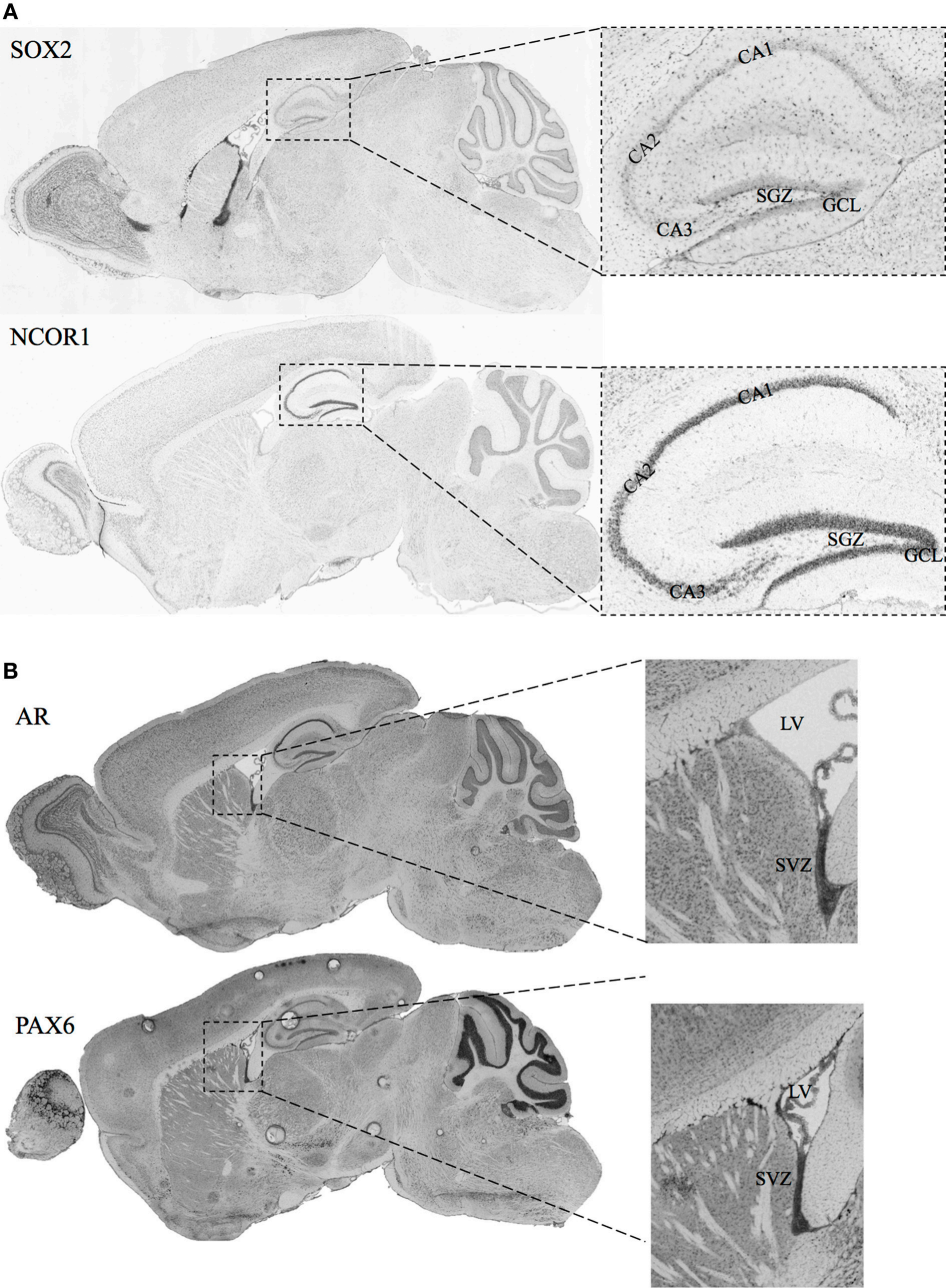
***In-situ* Hybridization images for selected candidate cell fate determinant pairs from Allen Brain Atlas. (A)** Images showing the staining for SOX2 and NCOR1 in the sagittal section of mice brain. The right panels show the zoomed view for the Lateral Ventricle (LV) and the SVZ. **(B)** Images showing the staining for AR and PAX6 in the sagittal section of adult male mice brain. The right panels show the zoomed view for the Lateral Ventricle (LV) and the SVZ. Original images can be accessed freely on http://mouse.brain-map.org/. (SOX2: http://mouse.brain-map.org/experiment/show/79677365, NCOR1: http://mouse.brain-map.org/experiment/show/687, AR: http://mouse.brain-map.org/experiment/show/100142554, PAX6: http://mouse.brain-map.org/experiment/show/79677341).

A necessary condition for the cell fate determinant pairs is that they are colocalized around the regions of neurogenesis (SGZ and SVZ) in adult male mice. Hence, we have included *in-situ* hybridization images for two of our candidate pairs (*Sox2*—*Ncor1* and *Ar*—*Pax6*) from Allen Mouse Brain Atlas as shown in Figures 3A,B. The details of the experiments and imaging can be found elsewhere (See Materials and Methods, *In-situ* Hybridization).

It is not surprising that stem cell marker *Sox2* is identified as a potential candidate in 6 out of 18 candidate pairs in SGZ to GCL region. Our candidate pair *Sox2*—*Srrt* predicted as cell fate determinant in SGZ is reported to function together as a fundamental mechanism to maintain neural stem cell identity in SVZ (Andreu-Agullo et al., 2011). The only *Sox2* candidate from the SVZ—OB analysis is the *Sox2*—*Foxo1* pair which is well characterized for having a pivotal role in maintaining stem cell identity (Zhang et al., 2011).

*Ncor1* is another factor predicted together with *Sox2* as a cell fate determinant in SGZ to GCL analysis. NCOR1 acts as a transcriptional co-regulatory protein, which recruits histone deacetylases to DNA promoter regions hence assisting nuclear receptors in the down regulation of DNA expression. Its expression in the mouse brain appears to be highly specific and localized around DG as observed from the *in-situ*-hybridization images from the Allen Mouse Brain Atlas (Data not shown). The interaction between NCOR1 and SOX2 is reported via the SMRT/NcoR complex during neurogenesis in NSC by Engelen et al. but requires functional identification on the cellular level (Engelen et al., 2011). The ISH of *Sox2* and *Ncor1* in Figure 3A indicates a highly specific *Ncor1* localization around DG whereas *Sox2* is localized around SGZ as well as in the SVZ, Lateral Ventricles (LV) and the Rostral Migratory Stream (RMS). These suggest significant colocalization of *Sox2* and *Ncor1* expression around SGZ and GCL *in-vivo*.

Our candidate pair *Eed*—*Hic1*, consists of Embryonic Ectoderm Development gene (*Eed*), a member of the epigenetic modifier complex Polycomb Repressive Complex 2 (PRC2), and an upstream transcription factor of Wnt signaling pathway, *Hc1* (Valenta et al., 2006). The canonical PRC2 comprises of *Eed*, *Suz12*, and *Ezh2* and controls stem cell development into different cell and tissue types of the body (Kaneko et al., 2010). PRC2 functions to methylate lysine 27 of histone 3 (H3K27me3) via interactions with the methyl transferase enhancer-of-zest 2 (*Ezh2*). PRC2 and *Ezh2* serve also in balancing self-renewal vs. differentiation and neuron vs. glial fate choices in early and late embryogenesis respectively (Pereira et al., 2010). Lately, HIC1 is demonstrated to recruit PRC2 for regulating the expression of a subset of its targets (Boulay et al., 2012) strongly suggesting that the HIC1—EED pair can function as a cell fate determinant via Wnt dependent manner.

Among the SVZ—OB cell fate determinant pairs we focus on *Ar*—*Pax6* pair. The transcription factor *Pax6* is reported to be essential for generating specific subpopulation of granule and periglomerular neurons in the olfactory bulb and neuronal and glial populations in the SGZ (Kohwi et al., 2005; Klempin et al., 2012). In Figure 3B, we demonstrate the localization of both *Ar* and *Pax6* around the SVZ. Furthermore, *Pax6*, and not *Ar*, expression is localized in OB.

AR has been associated as a potent regulator of adult neurogenesis only very recently. It has been reported by Hamson et al., that androgens, such as testosterone (T) and dihydrotestosterone (DHT), but not estradiol, increased the survival of new neurons in the DG and regulate hippocampal neurogenesis in an *Ar* dependent manner (Hamson et al., 2013). Here we identify a possible link between the *Pax6* and *Ar* in determining the balance between self-renewal and differentiation in the adult SVZ.

## Discussion

In this work we have focused on identifying region specific molecular mechanisms and cell fate determining factors in two main areas of neurogenesis (SGZ and SVZ) in the adult mammalian brain. To our knowledge, this is the first study attempting to identify genome wide differential regulatory mechanisms between two discrete regions of adult neurogenesis (SGZ and SVZ).

We have combined matching strain, sex and age mice gene expression profiles of (i) SGZ and GCL (Miller et al., 2013) with SVZ and OB (Ramos et al., 2013). We have transformed these region specific expression profiles to discrete values and integrated them forming the complete dataset.

Subsequently, we have constructed region specific GRNs via reported interactions in MetaCore and by carrying out network contextualization to each region. This allowed us to pinpoint region specific regulatory factors within these region specific GRNs, which are differentially active in each region. We have characterized *Hif1a*, *Nr3c1*, and *Ar* as three essential factors that are responsible for the differential regulation observed in these regions.

*Hif1a* is demonstrated to promote neurogenesis in a Wnt pathway dependent manner. HIF-1 is heterodimer of HIF-1α and HIF-1β/ARNT that regulates the response to hypoxia. *Hif1a* knockout, which encodes the HIF-1α subunit, reduces Wnt pathway target gene expression, actively reducing BrdU incorporation and the number of newborn *Dcx* positive neurons in the SGZ of mammalian hippocampus. These effects were rescued by the inhibition of GSK-3 and by expression of stabilized β-catenin, suggesting the HIF-1 involvement in the upstream of the Wnt pathway to promote neurogenesis (Mazumdar et al., 2010).

Furthermore, differential regulation of *Hif1a* (up-regulated in SGZ) arguably has unexpected implications such as promoting diverse niches within SGZ and SVZ with varying oxygen sensitivity and Wnt/β-catenin activity. The recent finding from Christen et al., demonstrate that the oxygen saturation in the adult rat brain is highly heterogeneous and can be detected with high spatial resolution via magnetic resonance imaging (MRI) (Christen et al., 2014). This brings us to the conclusion that oxygen saturation/sensitivity is one of the factors giving rise to the differences observed between SGZ and SVZ neurogenesis. Therefore, further *in-vivo* studies addressing oxygen dependency of SGZ and SVZ neurogenesis are required.

*Ar* (androgen receptor) and *Nr3c1* (glucocorticoid receptor) belong to a family of nuclear hormone receptors of the NR3C class, which also includes mineralocorticoid and progesterone receptors. They are ubiquitously expressed in various tissues and regulate downstream gene expression by binding to the nuclear response elements of the genome or direct interaction with other TFs such as *NF-kappaB*, *Ap-1* or *Stat*.

Hamson et al. recently demonstrated that gonadal steroids [testosterone (T) and dihydrotestosterone (DHT)] are regulating adult neurogenesis in adult male rats. They have provided evidence that androgens promote neurogenesis in the adult hippocampus by increasing the survival of newborn neurons via an Ar-dependent mechanism (Hamson et al., 2013). *Nr3c1* is a novel prediction resulted from our analysis and awaiting to be experimentally validated with adult neurogenesis.

In addition to region specific differences, an important conclusion from our analysis indicates the gender specificity of adult neurogenesis. Our analyses have been performed on male adult mice. The pivotal role of *Ar* in SGZ and *Nr3c1* in SGZ sub-networks highlight the significance of niche specific expression of nuclear hormone receptors in the neurogenesis context. Considering gender dependent expression of *Ar* and *Nr3c1*, *our results strongly indicate gender as well as region specificity of neurogenesis in the adult mammalian brain*. Further studies are required to address gender and region specificity of adult neurogenesis. Focusing on region specific spatial structures with quantification of hormones in each niche would unveil the intrinsic properties of adult SGZ and SVZ NSCs, their impact on aging and their potential to regenerate lost cell populations in neurodegenerative diseases or brain injuries.

Lastly, we have performed an in-depth analysis aiming to elucidate the region specific cell fate determinant pairs in SGZ and SVZ. A significant and unique contribution in this work is the developed methodology that systematically determines TF combinations (pairs) of cell fate determinants following an *unbiased in-silico* analysis. The methodology we have implemented considers NSCs (in this study) as the source and the differentiated cell as the target phenotype without prior knowledge to determine a list of cell fate determinant pairs with statistical significance. Henceforth, the scope of this study can easily be extended to other neural cell lineage differentiations such as the differentiation of astrocytes from the TAPs of the SGZ or the dopaminergic neurons of the OB from the SVZ.

Finally, using our methodology we have identified 18 TF pairs in SGZ to GCL and 13 TF pairs in SVZ to OB as cell fate determinant candidates. We present all 31 pairs in two NSCs regions; most of them having at least one gene associated with neurogenesis. In all but one (*Sox2*—*Srrt*) of the predicted pairs the interaction between the genes in the pair and the function of the predicted pair as a cell fate determinant remains to be experimentally verified.

In SGZ to GCL, 13 out of 18 pairs have at least one gene associated with neurogenesis/differentiation whereas; one pair is reported to maintain neural stem-cell identity in SVZ (*Sox2*—*Srrt*) (Andreu-Agullo et al., 2011). The remaining five pairs constitute novel predictions resulted from our study. Nine out of thirteen pairs predicted in SVZ to OB, have at least one of the genes (two pairs have both genes) is linked to neurogenesis/differentiation. The remaining four pairs are novel predictions that are not yet associated with neurogenesis. We would like to specifically emphasize the pairs (*Sox2*—*Ncor1*) and (*Id4*—*Tcf3*) in SGZ to GCL and (*Ar*—*Pax6*) and (*Egr2*—*Lhx2*) in SVZ to OB that have been predicted to function as cell fate determinants.

In summary, we characterized two aspects of adult neurogenesis: site-specific regulatory mechanisms in SGZ vs. SVZ and candidate cell fate determinant factors for each region. We identified putative factors and target genes, many of which are involved in NSC identity and maintenance. We demonstrate here possible gender and niche-derived differences based on cell surface and nuclear receptors via *Ar*, *Hif1a* and *Nr3c1*. We have reported 31 candidate cell fate determinant pairs, ready to be validated, for SGZ and SVZ together. We focused on cell fate determination by AR—*Pax6* in SVZ and *Sox2*—*Ncor1* in SGZ. Both pairs are expressed and localized in the suggested anatomical structures as shown by the ISH and found to physically interact. An extrapolation of our results suggests that the common features of neurogenesis observed in the NSC populations in SGZ and SVZ should be inspected very carefully. The fact that many cell surface and nuclear receptors have differentially regulatory roles indicates strong involvement of the niche-derived factors. Hence, to be able to understand neurogenesis in depth we will have to develop gender and region specific animal models of neurogenesis which constitute the prerequisite for studying gender bias observed in neurodegenerative diseases and aging.

## Author contributions

Gökhan Ertaylan contributed to the study design, collected data, performed the analysis and drafted the manuscript. Satoshi Okawa helped perform the analysis and helped draft the manuscript. Jens Christian Schwamborn discussed findings and interpretation of the results and helped drafting the manuscript. Antonio del Sol designed the study, discussed findings and interpretation of the results and drafted the manuscript.

### Conflict of interest statement

The authors declare that the research was conducted in the absence of any commercial or financial relationships that could be construed as a potential conflict of interest.

## Abbreviations

BrdU: 5′-bromodeoxy-uridine
BG: Background
BMP: Bone Morphogenetic Protein
CNS: Central Nervous System
DNA: Deoxyribonucleic Acid
EGF: Epidermal Growth Factor
GCL: Granule Cell Layer
GFAP: Glial Fibrillary Acidic Protein
IL: Interleukin
LIF: Leukemia Inhibitory Factor
INF: Interferon
LPS: Lipopolysacharide
LV: Lateral Ventricles
NF-κB: Nuclear Factor kappa B
NPC: Neural Progenitor Cell
OB: Olfactory Bulb
RMS: Rostral Migratory Stream
RNA: Ribonucleic Acid
SCC: Strongly Connected Component
SGZ: Subgranular Zone
Shh: Sonic Hedgehog
SOX: Sry-containing
SVZ: Subventricular Zone
TNF: Tumor Necrosis Factor
Wnt: Wingless-type
MMTV: integration site family member.
