# Supplementary material for: Gene regulatory network analysis reveals differences in site-specific cell fate determination in mammalian brain
Source: Front Cell Neurosci. 2014 Dec 18;8:437. doi: 10.3389/fncel.2014.00437 (PMC4270183; doi:10.3389/fncel.2014.00437)
Supplement: Supplementary file 2 [file DataSheet1.DOCX]

***Supplementary Material***

**Gene Regulatory Network Analysis Reveals Differences in Site-specific Cell Fate Determination in Mammalian Brain**

**Gökhan Ertaylan ^1^, Satoshi Okawa ^1^, Jens Christian Schwamborn ^2^, Antonio del Sol ^1^***

^1^Computational Biology, University Luxembourg, Luxembourg Centre for Systems Biomedicine, 7 avenue des Hauts Fourneaux, 4362 Belval, Luxembourg

^2^Developmental and Cellular Biology, University Luxembourg, Luxembourg Centre for Systems Biomedicine, 7 avenue des Hauts Fourneaux, 4362 Belval, Luxembourg

*** Correspondence:**

Antonio del Sol ([antonio.delsol@uni.lu](mailto:antonio.delsol@uni.lu))

Luxembourg Centre for Systems Biomedicine, 7 avenue des Hauts Fourneaux, 4362 Belval, Luxembourg.

**Supplementary Data**

1. **Supplementary Figures and Tables**

## Suplementary Tables

The Supplementary-Tables.xlsx file includes seven tabs that have the data required to reproduce the work described in this study. First three tabs include boolean the Quartile Expression values of i) SGZ vs SGZ, ii) SGZ vs GCL, iii) SVZ vs OB. Tabs 4,5 and 6 are the gene regulatory interactions downloaded from Metacore describing SGZ vs SVZ, GCL vs 12CL (BG), OB vs 12CL (BG) respectively. These tables used to construct the networks in further analysis. Finally although it is not extensive, the “Genes Associated and Present” tab highlights some of thhe genes reported by our study and/or associated with neurogenesis earlier with their references.

## Suplementary Figures

The Supplementary-Figures includes three figures. Figure S1: SCC of the AR contextualized SGZ network, Figure S2: SCC of the AR contextualized SVZ network in degree sorted circle layout format and Figure S3: Methodology for determining candidate cell fate determinant pairs. This methodology is explained in methods in detail.

## Suplementary Networks

The Supplementary-Networks.cys consists of SGZ specific gene regulatory network and its SCC, SVZ specific gene regulatory network and its SCC, SGZ specific & AR contextualized gene regulatory network and SVZ specific & AR contextualized gene regulatory network.

1. **References^[[1]](#footnote-1)^**

Crespo, I., & Del Sol, A. (2013). A general strategy for cellular reprogramming: The importance of transcription factor cross-repression. Stem Cells (Dayton, Ohio), 31(10), 2127–2135. doi:10.1002/stem.1473

Crespo, I., Krishna, A., Le Béchec, A., & Del Sol, A. (2012). Predicting missing expression values in gene regulatory networks using a discrete logic modeling optimization guided by network stable states. Nucleic Acids Research. doi:10.1093/nar/gks785

Hsieh, J. (2012). Orchestrating transcriptional control of adult neurogenesis. *Genes & Development*, *26*(10), 1010–1021. doi:10.1101/gad.187336.112

Milenkovic, Dragan; Berghe, Wim Vanden; Boby, Céline; Leroux, Christine; Declerck, Ken; Szic, Katarzyna Szarc vel; Heyninck, Karen; Laukens, Kris; Bizet, Martin; Defrance, Matthieu; Dedeurwaerder, Sarah; Calonne, Emilie; Fuks, François; Haegeman, Guy; Haenen, Guido R. M. M.; Bast, Aalt; Weseler, Antje R. (2014): Significant pathways (MetaCore MetaCore Bioinformatics software from Thomson Reuters,https://portal.genego.com/) of the gene expression profiles of leukocytes after consumption of monomeric and oligomeric flavanols (MOF) derived from grape seeds involved in immunity, cell signalling and cell adhesion. Figure_2.tif. PLOS ONE. 10.1371/journal.pone.0095527.g002.

Miller, J. A., Nathanson, J., Franjic, D., Shim, S., Dalley, R. A., Shapouri, S., et al. (2013). Conserved molecular signatures of neurogenesis in the hippocampal subgranular zone of rodents and primates. *Development*, *140*(22), 4633–4644. doi:10.1242/dev.097212.

Ramos, A. D., Diaz, A., Nellore, A., Delgado, R. N., Park, K.-Y., Gonzales-Roybal, G., et al. (2013). Integration of Genome-wide Approaches Identifies lncRNAs of Adult Neural Stem Cells and Their Progeny In Vivo. *Cell Stem Cell*, *12*(5), 616–628. doi:10.1016/j.stem.2013.03.003.

1. Provide the doi when available, and ALL complete author names. [↑](#footnote-ref-1)
