# Supplementary material for: Gene regulatory network analysis reveals differences in site-specific cell fate determination in mammalian brain
Source: Front Cell Neurosci. 2014 Dec 18;8:437. doi: 10.3389/fncel.2014.00437 (PMC4270183; doi:10.3389/fncel.2014.00437)

Figure – S1

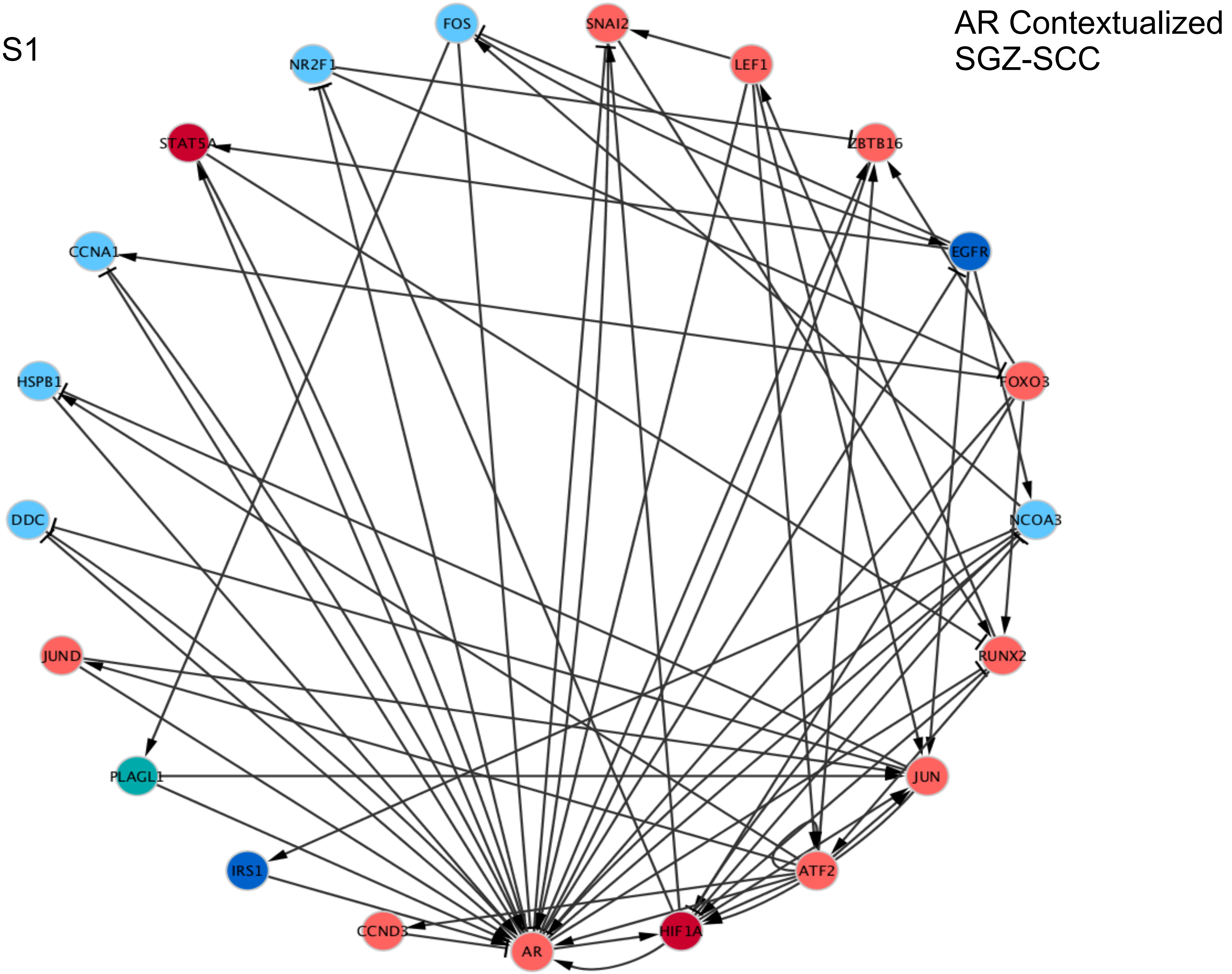

AR Contextualized
SGZ-SCC
FOS
SNAI2
LEF1
NR2F1
STAT5A
ZBTB16
EGFR
CCNA1
HSPB1
FOXO3
DDC
NCOA3
JUND
RUNX2
PLAGL1
JUN
IRS1
ATF2
CCND3
HIF1A
AR

Figure – S2

AR Contextualized
SVZ-SCC

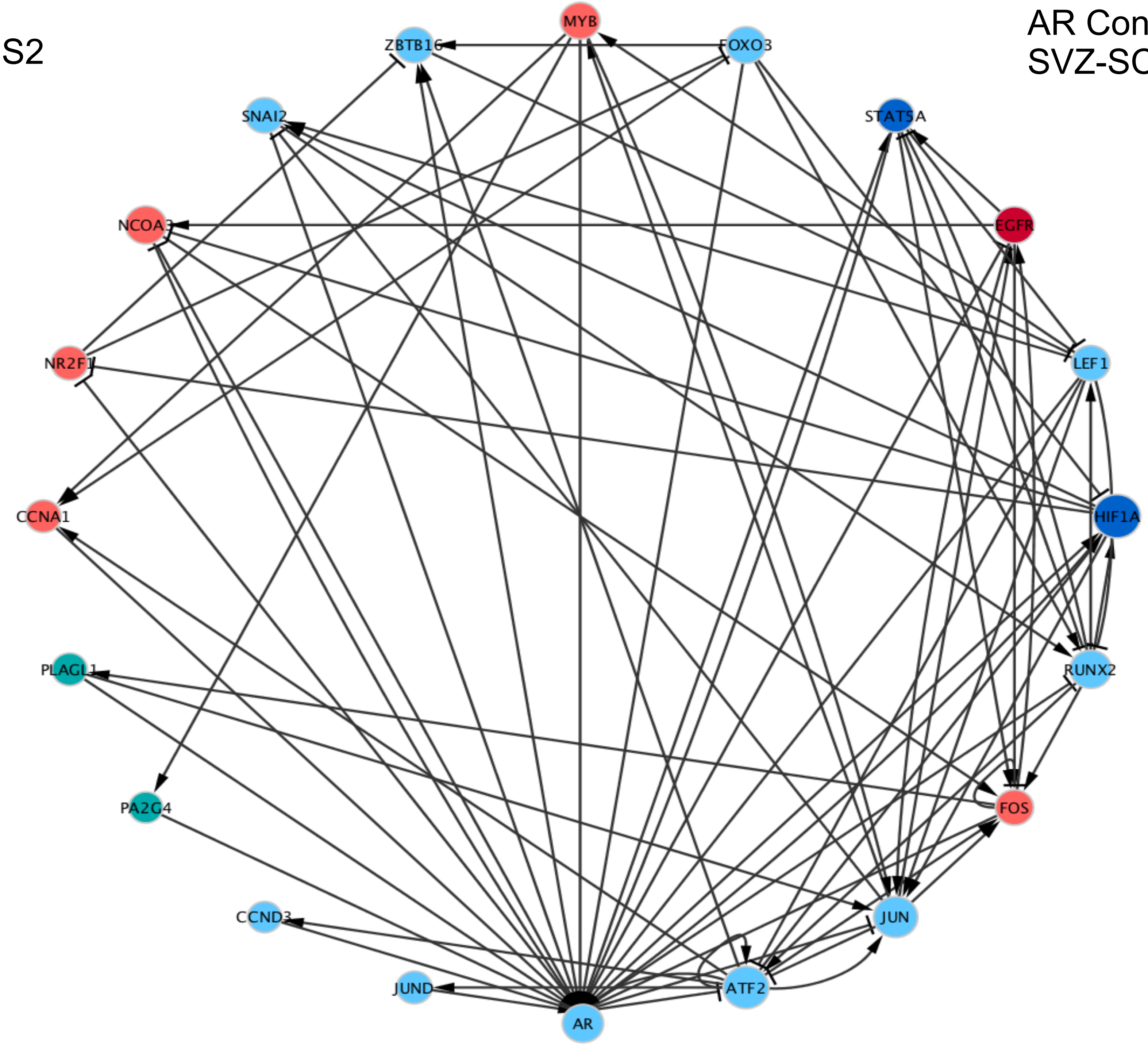

Figure – S3 Methodology for determining candidate cell fate determinant pairs

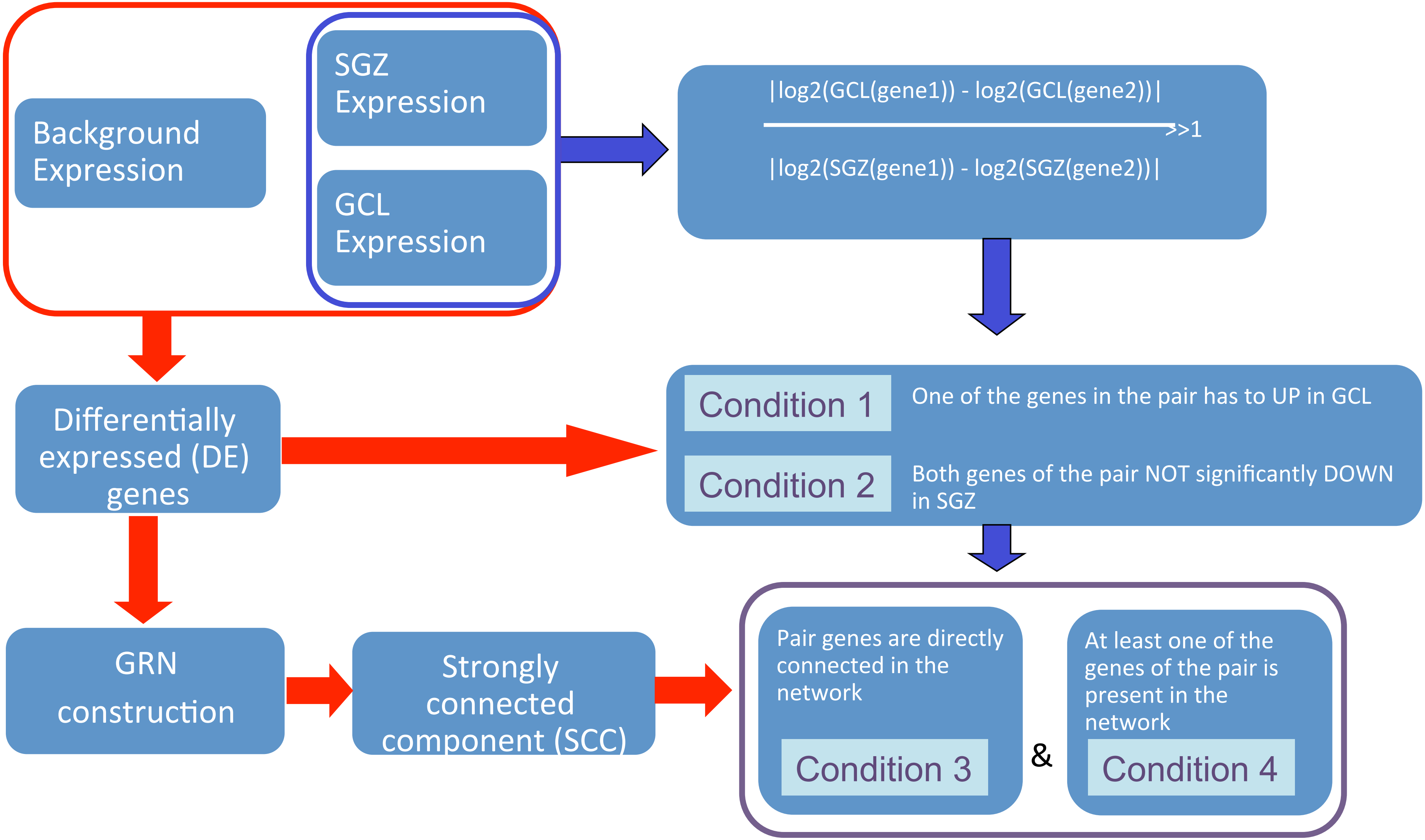

Supplement: Supplementary file 4 [file Image1.PDF]
